# Efficacy of clarithromycin on biofilm formation of methicillin-resistant *Staphylococcus pseudintermedius*

**DOI:** 10.1186/1746-6148-8-225

**Published:** 2012-11-21

**Authors:** Matthew DiCicco, Suresh Neethirajan, Ameet Singh, J Scott Weese

**Affiliations:** 1 School of Engineering, University of Guelph, Ontario, Canada; 2 Department of Clinical Studies, Ontario Veterinary College, University of Guelph, Ontario, Canada; 3 Department of Pathobiology, Ontario Veterinary College, University of Guelph, Ontario, Canada

**Keywords:** MRSP, *S*. *pseudintermedius*, Methicillin-resistant, Therapeutic, Bacteria, Biofilm, Macrolide, Clarithromycin

## Abstract

**Background:**

Surgical site infections (SSIs) caused by biofilm-forming methicillin-resistant *Staphylococcus pseudintermedius* (MRSP) have emerged as the most common hospital-acquired infections in companion animals. No methods currently exist for the therapeutic remediation of SSIs caused by MRSP in biofilms. Clarithromycin (CLA) has been shown to prevent biofilm formation by *Staphylococcus aureus*. This study aims to assess the *in vitro* activity of CLA in eradicating MRSP biofilm formation on various materials.

**Results:**

Quantitative assay results (P = 0.5126) suggest that CLA does not eradicate MRSP biofilm formation on polystyrene after 4 – 24 h growth periods. Scanning electron micrographs confirmed that CLA did not eradicate MRSP biofilm formed on orthopaedic implants.

**Conclusions:**

By determining the *in vitro* characteristics and activities of MRSP isolates alone and against antibiotics, *in vitro* models of biofilm related infections can be made. *In vitro* data suggests that CLA does not effectively eradicate *S*. *pseudintermedius* biofilms in therapeutic doses.

## Background

Surgical site infections (SSIs) are an inherent risk of any surgical procedure and can lead to morbidity, prolonged hospitalization, client frustration, frustration of medical caregivers, and increased treatment costs in veterinary medicine [1] and have been reported as a complication of 0.8% to 18.1% of operations in dogs and cats, depending on the surgical classification [2-4]. They are of particular concern with implanted biomaterials such as orthopaedic implants, suture material, and indwelling medical devices.

*Staphylococcus pseudintermedius* is an opportunistic pathogen that can be found on the skin or in the ears, oral cavity, intestinal tract or other non-sterile sites of a large percentage of healthy dogs [5,6]. In addition to being one of the leading causes of SSIs, staphylococci have a tendency to acquire resistance to antimicrobial agents [7]. Of particular concern is methicillin-resistance, which confers resistance to all beta-lactam antimicrobials. Methicillin-resistant *S*. *pseudintermedius* (MRSP) has rapidly emerged in companion animals and MRSP infections are being reported with increasing frequency in veterinary hospitals, becoming a major cause of pyoderma and SSIs in dogs [8-12]. MRSP infections are a tremendous concern in companion animals as they are challenging to eradicate being recalcitrant to traditional antimicrobial therapy, both due to their resistance to beta-lactam antimicrobials and because they typically have also acquired resistance to various other antimicrobial classes [1]. Reasons for the rapid emergence of MRSP are not well understood, however, one potential virulence factor that has received little attention in this bacterium is the ability to form biofilm. A bacterial biofilm is defined as a complex community of microorganisms embedded within a self-produced carbohydrate matrix attached to biological or non-biological surfaces [13].

Biofilm formation is an important virulence factor of methicillin-resistant *Staphylococcus aureus* (MRSA) implant-associated infections in humans [13]. It has been suggested that bacteria embedded within a biofilm enter a sessile state which is an important defense mechanism [14]. Biofilm-embedded bacteria are encased in a self-produced extracellular polysaccharide layer which protects against host immune responses, shear forces and antimicrobial penetration. Infections caused by biofilm-forming bacteria pose a tremendous challenge and can be difficult to control due to these protective mechanisms. Bacteria living within biofilms may be highly resistant to antimicrobials that are effective against their free-living counterparts as the minimal inhibitory concentration (MIC) for sessile bacteria within biofilms can be 10 to 1000 times as high as their planktonic form [15,16]. Biofilm formation has been hypothesized as one of the reasons for the emergence of a few successful MRSP clones internationally [17,18].

Bacterial biofilms may be of particular concern in veterinary orthopaedic surgery associated with implants [19]. Implants are a risk factor for MRSP SSIs [12] and implant associated infections can be difficult to control—perhaps in large part because of biofilm formation. Therapeutic options available to treat biofilm-associated infections are therefore limited, and removal of infected orthopaedic devices, with the associated morbidity and treatment costs, may be the only viable option [20]. Further development of alternative treatment regimens for biofilm-associated infections is needed.

Clarithromycin (CLA), a macrolide antimicrobial, has been shown to have potent *in vitro* and *in vivo* anti-biofilm activity against *S*. *aureus* and *Pseudomonas aeruginosa* alone and in combination with other antimicrobials, independent of its inherent antimicrobial activity [13,21-26]. This suggests that CLA could be an option for prevention or eradication of MRSP biofilms. However, biofilm formation (and presumably the factors that regulate biofilm formation) varies between bacterial species, and these factors have not been investigated for MRSP. The objective of this study was to determine the inhibitory effect of clarithromycin on MRSP biofilm formation using a microtiter plate assay.

## methods

### Bacterial isolate screening

30 epidemiologically unrelated MRSP isolates from dogs from different geographic regions were screened for biofilm production via microtiter plate assay (MPA) [27]. Briefly, overnight cultures were suspended in 5 ml of tryptic soy broth (TSB) supplemented with 1% glucose to achieve a turbidity equivalent to a 0.5 McFarland standard (~10^8^ CFU/ml). 200 μl of each inoculum was transferred in triplicate to a 96-well polystyrene microtiter plate and incubated under aerobic conditions for 24 h at 35°C. Following incubation, the plates were washed three times with phosphate buffered saline (PBS) to remove non-adherent cells and then heat fixed at 60°C for 60 minutes. Adhered cells were dyed with 0.1% (w/v) of crystal violet for 15 minutes and air dried at room temperature. After resolubilization with 95% ethanol, optical density (OD) reading of each well of the microtiter plate was assessed, taken at 570 nm (OD_570_). Readings of replicates for each isolate were averaged and subtracted from the OD_570_ reading of the negative control (wells containing uninoculated culture medium). OD_570_ was used as indication of biofilm production. Isolates were classified as biofilm producers if OD_570_ was >0.200 and further classified as strong, moderate, weak, or zero biofilm formers based on their final OD_570_ reading [28].

### Bacterial biofilm evaluation

Twenty MRSP isolates that were CLA resistant by Kirby Bauer disk diffusion and classified as biofilm producers (net OD_570_ > 0.200) were chosen for further study. Isolates resistant to CLA were chosen to ensure that any impact of CLA on biofilm formation was independent of antibacterial activity. Isolates were further characterized by sequence analysis of the mec-associated direct repeat unit (dru typing), with dru repeats and types assigned by the dru-typing.org database ( http://www.dru-typing.org/search.php). The impact of CLA on biofilm was assessed by MPA by comparing biofilm production in tryptic soy broth (TSB) supplemented with 1% glucose and TSB with 1% glucose plus 8 μg/ml CLA, as described above. To assess biofilm formation and the effect of CLA over time a previously screened high-biofilm forming isolate was chosen and 10 biological replicates assessed at 4, 8, 12, 16 and 24 h using the methods described above.

### Statistical methods

A student’s t-test was performed to compare groups with a P < 0.05 being considered significant. Statistical analysis was performed on commercially available software (SAS 9.2 TS Level 2M3; SAS Institute Inc., N.C., U.S.A).

### SEM protocol

Scanning electron microscopy (SEM) was used to examine the effect of CLA on MRSP adherence and biofilm production on orthopaedic bone screws. Briefly, an overnight culture of a high biofilm producing isolate of MRSP was inoculated into TSB with 1% glucose and TSB with 1% glucose + 8 μg/ml CLA. 316 LVM stainless-steel 20 mm orthopaedic bone screws (Veterinary Orthopaedic Implants, St. Augustine, FL, USA) were added to 5 ml of a 0.5 McFarland standard suspension of MRSP with and without CLA and incubated for 24 hours aerobically at 35°C. The screws were qualitatively evaluated at 4, 8, 12, 16, and 24 h of incubation. Each screw was washed with PBS, fixed at room temperature with 2.5% glutaraldehyde for 24 h and rinsed in Sorensen’s phosphate buffer three times for 15 min each. The screws were post-fixed with 1% osmium tetroxide for 30 min at room temperature, washed in Sorensen’s phosphate buffer twice for 15 min each, passed through an ethanol gradient (50%, 70%, 80%, 90%, and 99.5%), critical-point dried and finally sputter coated with gold. Screws were then imaged using a Hitachi S-570 scanning electron microscope at varying magnification and angles. Images were used to qualitatively assess and preliminarily compare bacterial adherence and the presence of adhered biofilm matrix.

## Results

**Figure 1:**
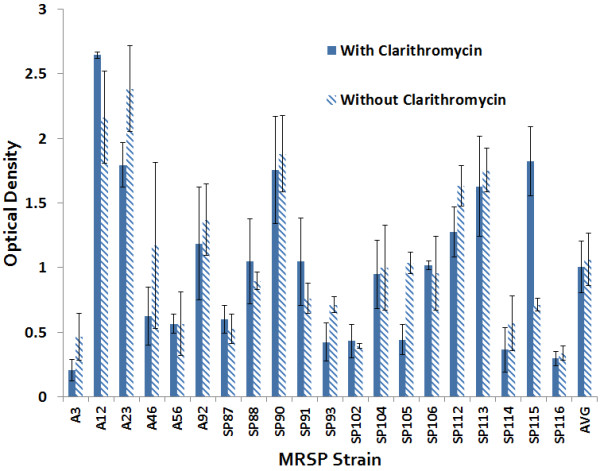
**Effect of Clarithromycin on MRSP after 24 h.** Biofilm forming potential of 20 MRSP strains and the effect of clarithromycin after 24 h as revealed by Crystal Violet Microtiter Assay. Mean OD_570_ +/− SD of the MRSP isolates with and without clarithromycin were 1.0 +/− 0.63 and 1.1 +/− 0.59, respectively. Clarithromycin had no significant effect (P = 0.5126) on MRSP biofilm formation.

**Figure 2:**
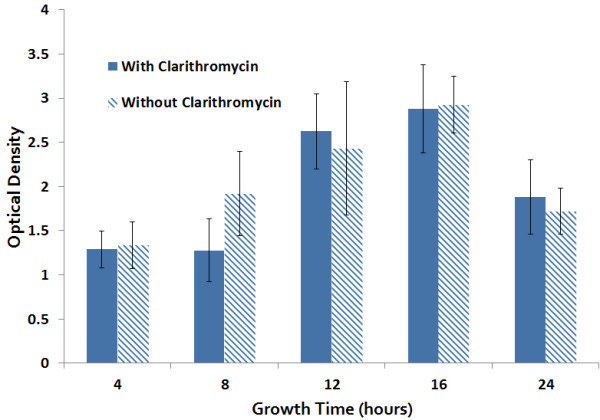
**Effect of Clarithromycin on MRSP over time.** Biofilm forming potential of MRSP A12 strain and the effect of clarithromycin over time as revealed by Crystal Violet Microtiter Plate Assay. Clarithromycin had no significant effect on MRSP biofilm formation between 4 and 24 h.

The twenty CLA-resistant isolates had OD_570_ readings ranging from 0.206 to 2.64 (Figure 1). There were 3 different *dru* types, corresponding to the two main international MRSP clones (Table 1) [29]. Of the twenty selected isolates 15%, 35%, and 50% were categorized as having strong, moderate, or low biofilm adherence properties, respectively (Table 1). There was no impact of CLA on MRSP biofilm formation on polystyrene, with a mean OD_570_ +/− SD of the 20 MRSP isolates with and without CLA of 1.0 +/− 0.63 and 1.1 +/− 0.59, respectively (P = 0.5216). Biofilm formation by high biofilm forming strain, MRSP A12, was evident by 4 hours of incubation and increased over time until 18 h (Figure 2).

**Table 1:** Origin, biofilm adherence classification, direct repeat unit (*dru*) and sequence type of study isolates

**Isolate selected**	**Adherence capabilities**	***Dru *typing**	**Sequence type**	**Origin location**
A3	LOW	9a	71	U.S.A
A12	STRONG	10h	68	U.S.A
A23	STRONG	10a	68	U.S.A
A46	MODERATE	9a	71	U.S.A
A56	LOW	9a	71	U.S.A
A92	MODERATE	9a	71	U.S.A
SP87	LOW	9a	71	Canada
SP88	LOW	9a	71	Canada
SP90	STRONG	9a	71	Canada
SP91	LOW	9a	71	Canada
SP93	LOW	9a	71	Canada
SP102	LOW	11a	68	Canada
SP104	MODERATE	10h	68	Canada
SP105	MODERATE	10h	68	Canada
SP106	MODERATE	9a	71	Canada
SP112	MODERATE	9a	71	Canada
SP113	MODERATE	9a	71	Canada
SP114	LOW	9a	71	Canada
SP115	LOW	9a	71	Canada
SP116	LOW	9a	71	Canada

*Dru* typing of chosen biofilm forming isolates of MRSP reveals a representative population of strains commonly found in veterinary hospitals across North America. Adherence capabilities were determined based on the model developed by Stepanovic *et al*., 2000.

**Figure 3:**
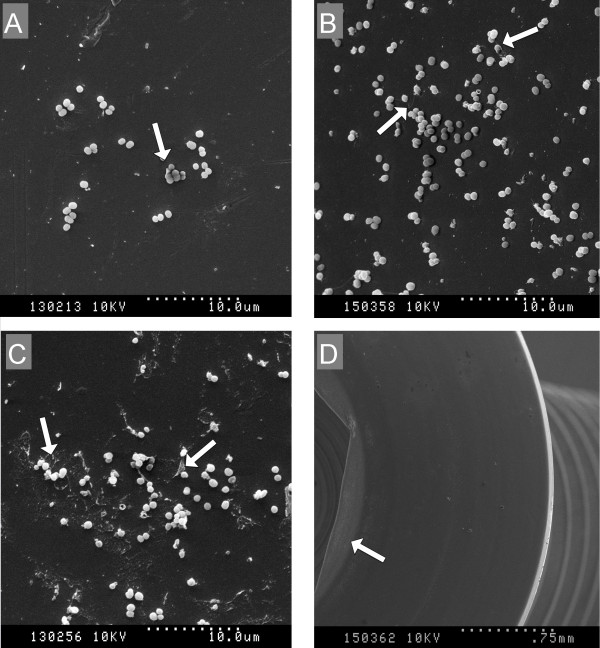
**SEM images of biofilms formed on canine orthopaedic bone screws treated with addition of clarithromycin.** (**A**) Showing minimal MRSP biofilm production at 4 h (**B**) showing increased EPS (extracellular polymeric substance) and morphological changes characteristic to biofilms at 12 h (**C**) firm attachment of MRSP biofilm, further increase of EPS and clumping of cells at 24 h (**D**) MRSP biofilm growth concentrated to specific areas on the screw head.

**Figure 4:**
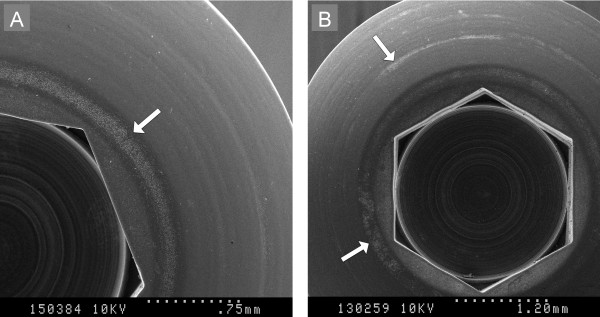
**SEM images of biofilm adherence around circular striations from machining on canine orthopaedic bone screws.** (**A**) MRSP biofilm growth at 8 h without CLA addition (**B**) MRSP biofilm growth at 24 h without CLA addition.

Qualitative evaluation of micrographs produced by SEM of surgical 316 LVM orthopaedic bone screws revealed the ability of MRSP to form biofilm on the surface of and between the screw threads. Adherent bacteria were evident by 4 h of infection with exopolysaccaride in variable amounts (Figure 3). Visually, CLA did not appear to inhibit MRSP adherence and biofilm formation. Non-homogenous biofilm formation was evident, with focal biofilm accumulation and circular deposition of biofilm evident on screw heads (Figure 4).

## Discussion

The ability of MRSP to form biofilm may be an important virulence factor and while it is closely related to MRSA, it appears that there are important cross-species differences. While biofilm production was common amongst this collection of MRSP isolates from different major clones and geographic regions, there was no evidence that CLA inhibits biofilm formation, in contrast to previous reports on MRSA [13,22,23]. Accordingly, results do not support the use of CLA for prevention of biofilm formation, and since there was no significant impact on biofilm formation regardless of classification, it is unlikely that CLA would have any impact on biofilm eradication.

CLA resistant strains of MRSP were chosen for this study to ensure that any impact of CLA was from its anti-biofilm effect, not simply from inhibition of bacterial growth. The Clinical and Laboratory Standards Institute interpretive breakpoint for clarithromycin resistance (≥ 8 μg/ml) [30] was chosen to represent a breakpoint concentration that is readily achieved through *in vitro* studies. One cannot exclude the possibility that an impact might have been present with higher concentrations of CLA, but the clinical relevance would be questionable.

The mechanism of biofilm prevention by macrolides seen in MRSA and other bacteria is not completely understood but current studies speculate that they also act through modification of the immune system’s inflammatory response to infection, and/or through a direct effect on bacterial virulence [24,26,31]. For this reason it cannot be excluded that CLA may be effective *in vivo*, however *in vitro* studies involving MRSA still support a preventative effect on biofilm formation [13]. It has also previously been shown that macrolide antibiotics affect quorum sensing—the initial mechanism behind bacterial biofilm formation and cell-cell communication—within the biofilm leading to reduced polysaccharide synthesis and instability of biofilm architecture [20,25,32].

It is possible that CLA does not impose a preventative biofilm forming mechanism on MRSP, as seen in MRSA, due to genetic variances not yet revealed between the two species. Currently, *ica* is considered to be the major operon responsible for staphylococcal biofilm formation [33] but its study in MRSP strains has not been performed. Alternative pathways for quorum sensing could also cause the mitigation of the previously demonstrated effect of macrolides. *Dru* typing results suggest a varying geographic distribution and representative chosen isolate population across the two current internationally predominant MRSP strains, ST68 and ST71 (Table 1) [34]. From this we can infer that genetic differences and therefore the effect of CLA on different strains of MRSP are likely minimal.

Time assessed biofilm development, while only one isolate was studied, suggests that biofilm formation occurs rapidly *in vitro*, since adherent bacteria and exopolysaccaride matrix were evident within 4 h by both the crystal violet MPA and through qualitative SEM evaluation of growth on surgical screws. Objective assessment of the impact of CLA on biofilm formation on screws was not possible since only one isolate was studied in a qualitative manner, yet these subjective data are in support of the crystal violet MPA and provide further evidence of a lack of efficacy of CLA for prevention of biofilm formation. The irregular biofilm patterns on screws, most notably the circular biofilm accumulations on screw heads, is consistent with preferential biofilm adhesion to invisible surface defects or irregularities in the machining process. This suggests that minor surface alterations, either from inherent defects or damage to implants during placement, could facilitate biofilm attachment *in vivo*.

*In vitro* evaluations of CLA on MRSP biofilm formation were performed on polystyrene and one orthopedically relevant biomaterial providing potential limitations to this study. Although no significant inhibitory effect of CLA on these materials was found, material properties of biofilm attachment sites could play a minor role in the susceptibility to and persistence of staphylococci infections [33]. Combinational therapy with CLA and varying antimicrobials has also been shown to have appreciable effects against MRSA biofilm formation [13,22,23]. Because of the potential benefit to biofilm formation prevention and the safety of macrolides shown in long-term randomized macrolide therapy, further study and use of CLA in combinational therapy and on varying biomaterials is recommended [20].

Large variances in both the amount of bacteria and antimicrobial in suspension in the 200 μl sample before addition to the microtiter plate could add to uncertainty in crystal violet microtiter plate assay results as previously described [27]. Optical determination of 0.5 McFarland and quick doubling-time of *S*. *pseudintermedius* could also contribute to the large standard deviations found for each averaged isolate OD_570_ reading. Microtiter plate washing techniques, as described in Stepanovic et al., also leave room for interpretation and could lead to the removal of transient bacterial biofilms, further adding to the variance in quantitative results for each isolate. Although the two currently most prevalent MRSP strains internationally were represented (ST68 and ST71) other biofilm forming strains of MRSP susceptible to the biofilm prevention mechanism in CLA might exist. Finally, the SEM study only accounts for one of 20 screened isolates across 11 biological replicates. Though the analysis was only for comparison the images are not representative of strains seen in the MPA.

## Conclusions

Results suggest that CLA does not inhibit MRSP biofilm formation on polystyrene, independent of the antimicrobial activity when evaluated through a crystal violet assay. Qualitative SEM imaging results also suggest that adhesion and formation of MRSP biofilms begin within 4 h of infection on stainless-steel with no inhibitory effect by CLA. However, CLA may inhibit MRSP biofilm on other surfaces such as titanium based implants. *In vivo* and additional *in vitro* studies evaluating the effect of CLA alone and in combination with other antimicrobials on MRSP biofilm formation through crystal violet assays and other biomaterials are warranted.

## Competing interest

All authors declare that they have no competing interests.

## Authors’ contributions

MD conducted the microtitre plate assay experiments, acquired SEM images and drafted the manuscript. SN, AS, and SW contributed in the conceptual design of the experiments. All authors read and approved the final manuscript.
